# Allegiance and Treatment Quality as Moderators of the Comparative Effectiveness of Psychotherapy? A Systematic Review and Meta-Analysis of Studies Comparing Humanistic Psychotherapy to Other Psychotherapy Approaches

**DOI:** 10.32872/cpe.9709

**Published:** 2025-02-28

**Authors:** Olivia Schünemann, Alessa Jansen, Ulrike Willutzki, Nina Heinrichs

**Affiliations:** 1 Institute of Psychology, TU Braunschweig, Braunschweig, Germany; 2 Bundespsychotherapeutenkammer, Berlin, Germany; 3 Department for Psychology and Psychotherapy, Witten/Herdecke University, Witten/Herdecke, Germany; 4 Department of Psychology, Bielefeld University, Bielefeld, Germany; Philipps-University of Marburg, Marburg, Germany

**Keywords:** RCT, allegiance, bona fide, study quality, adherence, humanistic psychotherapy

## Abstract

**Background:**

Achieving positive outcomes in comparative RCTs examining psychotherapy interventions may be moderated by other factors than treatments alone, namely allegiance and treatment quality (bona fide, adherence). Using the study sample of a recent comprehensive review on humanistic interventions by the German Scientific Board of Psychotherapy, we assumed that higher allegiance towards non-humanistic approaches and lower treatment quality in the humanistic intervention arm would result in worse outcomes for the humanistic groups.

**Method:**

We included studies in which a humanistic psychotherapy (sub-)approach was compared to another type of psychotherapy. Data was extracted independently by the authors. A priori defined meta-regression analyses were performed with allegiance and treatment quality as main moderators and study quality (risk of bias), type of active control, humanistic psychotherapy and target population (children/adolescents; adults) as exploratory.

**Results:**

The majority of studies showed non-allegiance towards humanistic intervention arms; only about half of the humanistic interventions were bona fide treatments demonstrating high percentages of potential biases in these comparative intervention studies. However, allegiance and bona fide were significant moderators only for two (allegiance) resp. one (bona fide) of five outcome comparison. Type of active control (cognitive behavioural therapy) and disorder group (anxiety disorders) emerged as further moderators.

**Conclusion:**

We found no clear evidence for allegiance or treatment quality impacting upon treatment outcome in this re-examination. Allegiance and treatment quality were not as relevant for outcomes in this meta-analysis of RCTs as expected.

## Highlights

- Analysed studies displayed substantial heterogeneity concerning allegiance and treatment quality.
- Only 10% of the studies showed allegiance in favour of HPT.
- Allegiance and bona fide were significant moderators in 2 (alleg.)/1 (bona fide) of 5 comparisons.
- Allegiance and treatment quality were not as relevant for outcomes as expected in our examination.

Humanistic psychotherapy (HPT) has been characterized as a psychotherapeutic approach focusing on “the conditions or stances by which people can come to intimately know themselves and, to the extent possible, to fulfill their aspirations” (Schneider & Leitner, 2002, p. 949). International literature lists HPT as one of the major psychotherapy approaches next to cognitive-behavioural, psychodynamic and systemic psychotherapy (e.g., Lambert, 2013). Like other psychotherapy approaches HPT is a broad and diverse psychotherapeutic approach embracing various subapproaches, for example client / person-centered psychotherapy (Rogers, 1961), constructivist psychotherapy (Neimeyer, 1995), emotion-focused psychotherapy (Greenberg et al., 1998), existential meaning making psychotherapy (Schneider & Krug, 2010), focusing-oriented psychotherapy (Gendlin, 1981), gestalt psychotherapy (Perls et al., 1994) and transpersonal psychotherapy (Wilber et al., 1986; selection suggested by Angus et al., 2015). Schneider and Längle (2012, p. 428) summarized the common assumptions of HPT as follows: “Humanism is concerned with such existential themes as meaning, mortality, freedom, limitation, values, creativity, and spirituality as these arise in personal, interpersonal, social, and cultural contexts. In psychotherapy, humanism places special emphasis on the personal, interpersonal, and contextual dimensions of therapy and on clients’ reflections on their relationship with self, others, and the larger psychosocial world”. Accordingly, all HPT subapproaches share the following principles: Endorsement of the centrality of an empathic and prizing therapeutic relationship and a focus on the promotion of client experiencing in the therapy process (Elliott et al., 2013). Nevertheless, significant differences between these subapproaches exist, e.g., in their preferred methods for building a therapeutic alliance, treatment indications, case conceptualizations or concepts for individual treatment planning (e.g., Elliott et al., 2021; GSBP, 2018).

A significant attempt to aggregate empirical results in the context of HPT is reflected in the meta-analysis by Elliott et al. (2013) who analysed 191 HPT-studies (through 2008) involving person-centered, supportive or nondirective, and other HPT subapproaches. Regarding pre-post effects of HPT (*n* = 191 studies with *n* = 14,235 clients), Elliott et al. (2013, p. 10) reported a weighted pre-post effect size of *d* = 0.93 with stable pre-follow up effects up to *d* = 1.11 when pooling effects of all subapproaches of HPT. Their comparison of HPT to other therapy approaches indicated no differences in pre-post results (weighted effect size *d* = 0.01; 95% CI [-0.05, 0.07]. However, the comparison group “other therapies” in this meta-analysis is not well described. Comparing HPT to cognitive-behavioural therapy (CBT) only, HPT was described as slightly inferior to CBT (*d* = -0.13, 95% CI [-0.21, -0.06]; Elliott et al., 2013, p. 10).

There are currently a number of psychotherapy approaches available for practitioners to deliver clinical services around Europe. In Germany, however, HPT is not part of the list accredited psychotherapists can choose from and be reimbursed for their services. To become part of this list, psychotherapy approaches have to undergo two independent evaluations in Germany: (1) a scientific evaluation conducted by the German Scientific Board of Psychotherapy (GSBP [Wissenschaftlicher Beirat Psychotherapie]); and – if they are evaluated positively by the GSBP – (2) subsequent assessment concerning cost-utility aspects (conducted by another, independent board). A recent evaluation of HPT by the GSBP on request of the Work Group Humanistic Psychotherapy (Arbeitsgemeinschaft Humanistische Psychotherapie, 2012) resulted in the conclusion that the approach is not evidence-based according to the Board’s criteria.

## Potential Reasons for These Discrepant Results: Allegiance and Treatment Quality

The discrepancy between the review result of the GSBP and other international reviews on HPT (e.g, by Elliott et al., 2013; Elliott et al., 2021) can be explained through the different methodological approaches, e.g. using meta-analyses and pooling effects for a certain disorder (Elliott et al., 2013; Elliott et al., 2021) vs. defining a required minimum of evidence (at least three RCTs with sufficient internal and external validity) for a certain number of diagnostic groups (currently: 4 out of 12) in the GSBP approach. Moreover, the discrepant definition of what constitutes HPT as defined by the German Work Group Humanistic Psychotherapy from the international definition, e.g. as defined in the reviews by Elliot is likely to be relevant here. The assignment of subapproaches to HPT is different across author groups and also within type of publications. Elliott et al. (2021) use the term HPT to embrace 10 subapproaches (person-centered therapy, emotion-focused therapy, motivational interviewing, gestalt, existential, psychodrama, focusing-oriented, expressive and body-oriented as well as “supportive / nondirective”), while in Elliott et al. (2013) the respective analyses included 9 subapproaches, omitting motivational interviewing. On the basis of current discussions in psychotherapy research (e.g., Wampold, 2015), we hypothesized in addition that two key reasons may have an impact on the effectiveness on HPT subapproaches (Schünemann et al., 2019):

1) Researchers’ allegiance that may be associated with a decrease of the (possible) effects of the non-preferred treatment condition (Munder et al., 2011), in case of the review of the GSBP a decrease of the effects of subapproaches of HPT. Leykin and DeRubeis (2009, p. 55) define “Allegiance, in the context of treatment outcome research, …[as] a belief in the superiority of a treatment. It usually also entails a belief in the superior validity of the theory of change that is associated with the treatment”. Often, this belief is associated with therapy outcomes and may reflect a risk of bias. A meta-analysis on the allegiance bias hypothesis demonstrated a moderate association between allegiance and treatment outcome moderated by methodological quality (Munder et al., 2011), indicating that allegiance as well as methodological quality may need to be taken into account for treatment comparisons (Munder et al., 2011). In the context of the HPT evaluation of the GSBP, researchers’ allegiance may have had an effect because many studies in the pool used a study design in which a humanistic subapproach was designed as control group in comparison to other psychotherapeutic approaches (e.g., CBT).

2) We further assumed that treatment quality of HPT studies in the GSBP evaluation may have an impact on outcomes. In this context, the two aspects of bona fide psychotherapy and adherence can be differentiated. Whereas bona fide represents *conceptual quality* of a treatment, adherence is rather concerned with *process quality*. For the present study, bona fide psychotherapy will be defined as follows: mentioning or describing an established psychological approach, psychological treatment principles, a treatment manual or active treatment ingredients (Benish et al., 2008) as well as the requirement that the intervention is implemented by a trained therapist (Wampold et al., 1997). Further, we use a definition of adherence in accordance with Munder et al.’s (2013) definition of treatment integrity: “[…] conceptualized broadly including adherence to specific treatment procedures (e.g., the importance of exposure in psychotherapy for post-traumatic stress disorder), common factors (e.g., therapeutic alliance), and therapist effects (i.e., differences in the effects due to individual therapists)” (Munder et al., 2013, p. 8). Concerning treatment quality, Wampold (2015) summarizes that trainers’ competence has only a small (*d* = 0.14) and adherence to protocol an almost negligible effect (*d* = 0.04) on treatment outcome. The (non-) effect of adherence to protocol may be explained with associated decreased patient-therapist alliance, increased likelihood of resistance to treatment and a lack of flexibility by the therapist. We hypothesized that the GSBP-evaluation included studies implementing HPT subapproaches (often as control group) in potentially fuzzy and poor quality so that significant HPT mechanisms could not work properly resulting in potentially decreased effects of the respective HPT subapproach.

## Objectives

The aim of this meta-analysis is the re-examination of studies included in the GSBP evaluation of HPT (GSBP, 2018) (based on the application of the Work Group Humanistic Psychotherapy and the additional searches conducted by the GSBP) to examine potential moderating effects of allegiance and treatment quality (bona fide psychotherapy and adherence) on the comparative effectiveness of HPT (including both efficacy and effectiveness studies). We examine whether in the HPT subapproaches vs. others psychotherapeutic interventions included in the GSBP evaluation 1) allegiance to a particular psychotherapeutic intervention and 2) treatment quality (bona fide/adherence) significantly moderate effect sizes. We expected higher allegiance towards other psychotherapy approaches and lower treatment quality to be significant predictors of lower effect sizes in the HPT condition.

## Method

### Design, Study Search and Procedure

This study was registered in the PROSPERO International prospective register of systematic reviews (CRD42019128983). The present full report relates to the second objective in this protocol (comparison of humanistic subapproaches versus other psychotherapeutic interventions).

The study pool which builds the basis of the present analysis is taken over from the GSBP (2018). For their final sample, the GSBP screened abstracts of *n* = 481 studies from which *N* = 114 went into the final pool of evaluated studies. The abstract screening procedure was based on 1) a list of studies submitted by the Work Group Humanistic Psychotherapy (*n* = 313), and 2) results from an independent literature search conducted by an independent institution. According to pre-specified PICOS criteria, all study abstracts were screened; the ones included went into full-text screening review by two independent reviewers (members of the GSBP) to decide about their further inclusion.

The present systematic review used the identical study pool of the GSBP (*N* = 114) in order to compare the results of this systematic review directly with the conclusions of the GSBP and by this to be able to provide recommendations for future analyses on psychotherapeutic approaches or methods by the GSBP.

**Figure 1:**
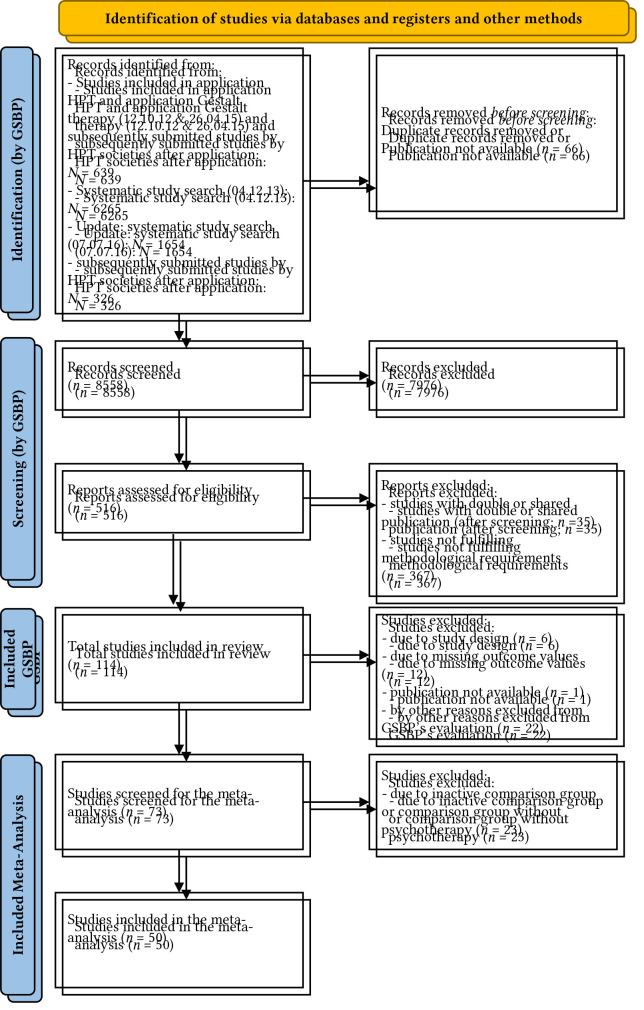
PRISMA Flow Diagram Showing the Process for Search and Selection of Studies

It was necessary to conduct a secondary study screening for eligibility because the present systematic review needs specific study data beyond the data relevant for the purpose of the GSBP. As result, we used *N* = 50 studies which compared HPT subapproaches to alternative evidence-based psychotherapeutic interventions (see flowchart Figure 1). The remaining studies were either excluded or relate to inactive control conditions.

Study characteristics for the present meta-analyses were extracted by one author (either OS or AJ). Study results, study quality, allegiance and treatment quality were rated by two of the authors independently (OS & NH or AJ & UW or NH & UW). Inter-rater reliability (Cohen`s Kappa) for adherence (only available for one of the two rater teams: NH, OS) across all intervention groups (IG and CG) was moderate (Altman, 1999): κ = .507 (*p* < .001); agreement = 76.7%. Inter-rater reliability (intra-class correlation, ICC) for allegiance (MARS index) was .91 (95% CI [.82, .95]; two-way mixed-effects ICC, absolute agreement, average for two coders, same rater team).

Discrepancies were first discussed within dyads and if discrepancies could not be clarified, were discussed among all four authors until a final decision was reached.

### Inclusion and Exclusion Criteria

Inclusion criteria were based on those of the GSBP and complemented by the availability of data we needed for the meta-analysis. Inclusion criteria were: 1) RCTs or non-randomized controlled trials with active control group and 2) report of pre- and post-assessments regardless of follow-up assessments. These criteria were complemented with the following exclusion criteria: 1) an active control group was missing (only two or more HPT subapproach-groups with no additional active control group precluding the examination of allegiance); 2) a metric outcome measure or post mean were lacking or 3) an indication of data manipulation could be found. For more details see Schünemann et al. (2019) or Supplementary Materials (*Additional inclusion and exclusion criteria*).

### Data Collection

We extracted information on participant characteristics, study characteristics, intervention characteristics, primary and secondary outcomes, risk of bias, allegiance, treatment quality (bona fide and adherence). Guidelines of the Cochrane Collaboration were used to estimate and substitute missing data for outcomes, e.g., calculating standard errors from exactly reported *t*-values. The primary effectiveness outcome was symptom severity at the end of treatment measured on a metric symptom specific scale. Outcomes on self-rating scales (e.g., BDI) were given priority over observer-rated scales (e.g., HDRS). As symptom reduction is not necessarily the primary target of change across different psychotherapy approaches, we extracted data for different outcome domains to analyse moderator effects specific to different outcome domains. Secondary outcomes were interpersonal outcomes (e.g., DAS), general assessment of functioning (e.g., GAF) and quality of life (e.g., WHO QOL). The primary outcome was extracted for short-term (end of intervention) and follow-up if available (6 months after end of intervention or the one closest to 6 months). As primary negative outcome drop-out until end of intervention was extracted.

### Assessment of Main and Exploratory Moderators: Study Quality, Allegiance and Treatment Quality

Allegiance was assessed according to the multilevel allegiance rating-scale (MARS) provided by Steinert et al. (2017). This instrument combines information about 1) researchers’ allegiance either respective treatment development or contribution to an etiological understanding of the treated disorder; 2) therapists’ allegiance; 3) trainers’ allegiance and 4) supervisors’ allegiance to a total score (0-4; Steinert et al., 2017).

Treatment quality with bona fide psychotherapy and adherence were rated using the definition by Wampold et al. (1997) and Benish et al. (2008) according to the following items: using/citing an established psychotherapy manual; used intervention is based on psychological principles; author mentions HPT (subapproach)/CG on own his/her website; author has other relevant HPT (subapproach)/CG publications; intervention was carried out by trained therapists. To meet the criterion of bona fide two of the items needed to be fulfilled. In the moderator analyses “bona fide” was compared to “non or unclear bona fide”. To fulfill the criterion of treatment adherence, treatment conditions and therapeutic procedure needed to be described in detail and adherence must have been proved by external raters. In the moderator analyses “non-adherence” was compared to “adherence”. Study quality of the included studies was rated according to the second version of Cochrane’s risk of bias tool (RoB 2.0; Higgins et al., 2018) considering the adaptions by Munder and Barth (2018) for its use in psychotherapy outcome research. Thus, the methodological quality was assessed via: 1) bias arising from the randomization process; 2) bias due to missing outcome data; 3) bias in outcome measurement; and 4) bias in selection of the reported result (Cochrane’s risk of bias tool) as well as 5) effect of adhering to intervention (see Higgins et al., 2018; Munder & Barth, 2018). For non-randomized controlled trials, the ROBIN-I tool (Sterne, Hernán, et al., 2016) was used to rate risk of bias. For moderator analyses a “low risk of bias” in each category was compared to an “unclear or high risk of bias”.

### Statistical Analyses

Standardized mean difference for metric measures and odds ratios for rare outcomes between the intervention groups at end of intervention and follow-up were calculated using the intention-to-treat sample, if available. For all analyses, a random effects model with inverse variance weights was applied (DerSimonian & Laird, 2015). Cochran’s Q-test was used and quantified using the I^2^-statistic (Higgins et al., 2003) to test statistical heterogeneity between study results for significance. Visual examination of funnel plots and Egger’s test (Sterne, Egger, & Smith, 2016) were applied to examine possible publication bias.

A priori defined subgroup (in case of categorical predictors) or meta-regression (in case of metric predictors) analyses (univariate) were conducted concerning study quality, allegiance, treatment quality, type of non-active control (waitlist vs. all others including TAU), type of HPT subapproach (client centered vs. all others) and population (children/adolescents vs. adults). Differences between subgroups were tested formally (Bucher et al., 1997; Deeks et al., 2001; Song et al., 2003). A posteriori (explorative) meta-regression analyses (univariate) were performed in case of considerable heterogeneity between studies for number of sessions, length of intervention, percentage of women, affective disorder, anxiety disorder, post-traumatic stress disorder (PTSD), F 54, study design (no RCT) and comparator (CBT). All analyses were conducted using the metafor package in R (Viechtbauer, 2010).

## Results

### Descriptive and Additional Results

**Figure 2:**
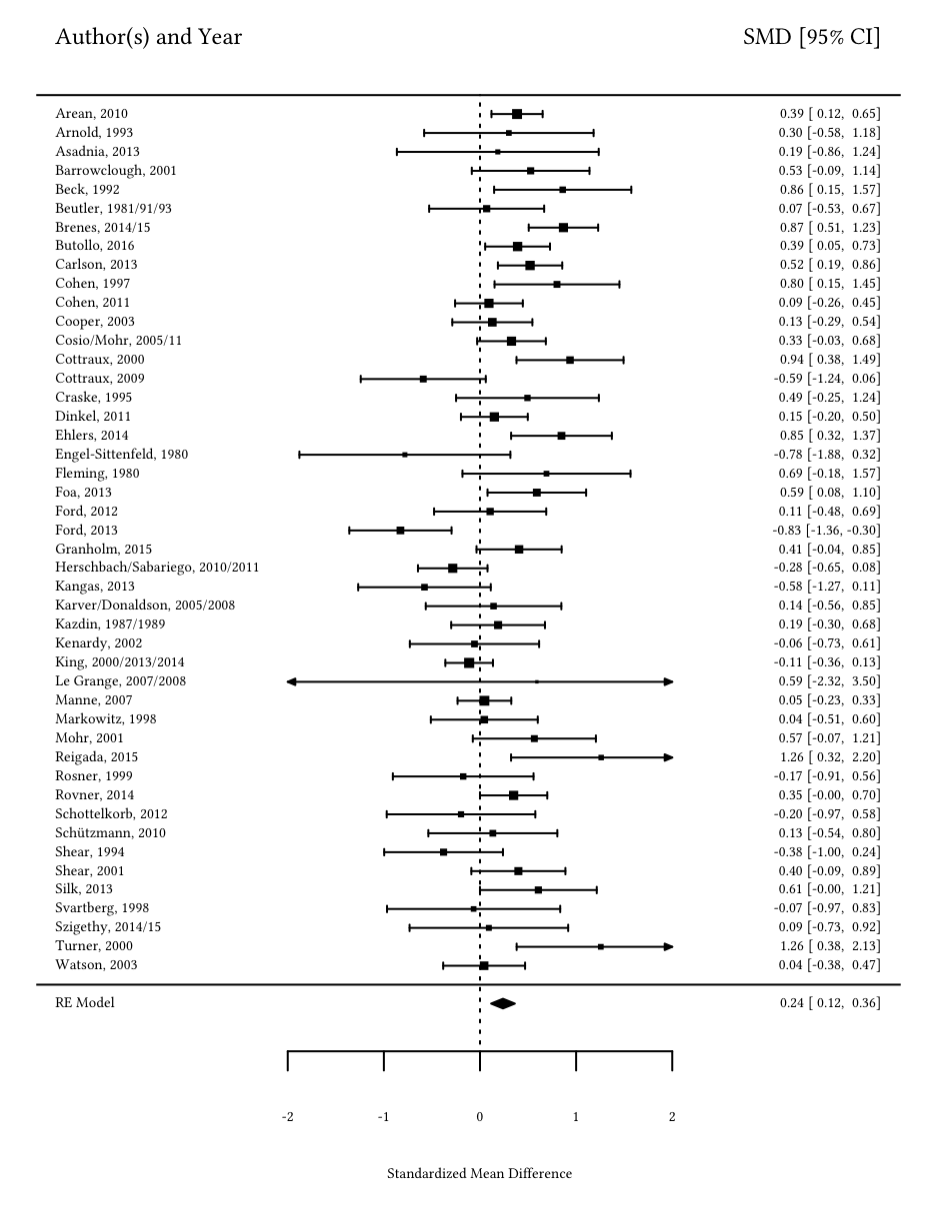
Exemplary Forest Plot of the Comparison HPT Approaches vs. Other Psychotherapy Only for the Primary Outcome Symptom Severity at End of Intervention

Because of space limitations additional information and results are documented in the Supplementary Materials. Individual characteristics of all studies are shown in the Supplementary Materials (Supplementary Table 1). All ratings for Cochrane’s risk of bias and ROBIN-I tool (study quality) as well as for allegiance and treatment quality for each study can be found in the Supplementary Materials (Supplementary Table 2). Further, the overall main effect sizes are presented in Supplementary Table 3. In order to avoid misunderstandings when interpreting the results of the overall effects a more detailed explanation of the main effects can also be found in Appendix C – Supplementary Information (see Preliminary Results: Overall main effect sizes of HPT). The forest plot for the primary outcome is displayed in Figure 2.

### Main Results: Univariate Moderator Effects – The Role of Allegiance and Treatment Quality

We will first report on moderation effects (pre-specified moderators followed by exploratory moderators) for post-intervention outcomes and then move to reporting the results for the same moderators at follow up.

The results for the moderator analyses for the symptom severity (primary outcome) are presented in Table 1A. No significant moderating effects on the primary comparative effectiveness outcome *symptom severity at end of intervention* could be shown for the pre-specified moderators study quality, allegiance or treatment quality.

**Table 1A:** Moderators Analysis for Primary Outcome: Symptom Severity at End of Intervention and at Follow-up

Moderators^a^	Symptom severity end of intervention (*N =* 47^b^)					Symptom severity at follow-up (*N =* 30^c^)				
	Intercept	β	*SE*	*p*	*R*^2^ in %	Intercept	β	*SE*	*p*	*R*^2^ in %
no or unclear allegiance against HPT	0.315	-.183	0.125	.144	6.84	**0.365**	**-.208**	**0.103**	**0.044**	**70.62**
non or unclear bona fide HPT	0.185	.116	0.127	.361	< 0.01	0.191	.159	0.118	.178	17.41
non or unclear bona fide CG	0.243	-.042	0.230	.854	< 0.01	0.300	-.241	0.235	.305	0.91
non adherence HPT	0.260	-.054	0.132	.679	< 0.01	0.294	-.018	0.125	.887	< 0.01
non adherence CG	0.226	.070	0.160	.673	< 0.01	0.240	.190	0.140	.175	8.27
low RoB RP	0.175	.111	0.128	.385	< 0.01	0.284	.001	0.126	.996	< 0.01
low RoB AI	0.210	.067	0.129	.602	< 0.01	0.369	-.208	0.124	.094	< 0.01
low RoB MI	0.194	.080	0.129	.537	< 0.01	0.228	.101	0.122	.407	< 0.01
low RoB MO	0.193	.065	0.140	.642	< 0.01	0.324	-.062	0.130	.637	< 0.01
low RoB SR	0.292	-.217	0.148	.142	< 0.01	0.286	-.008	0.165	.960	< 0.01
client centered psychotherapy	0.287	-.060	0.156	.698	< 0.01	0.302	-.022	0.156	.887	< 0.01
population: children	0.213	.128	0.158	.419	< 0.01	0.223	.280	0.143	.050	21.79
age*^d^	0.239	< .001	0.003	.932	< 0.01	0.493	-.006	0.004	.127	19.60
% of women*^d^	0.257	< .001	0.003	.867	< 0.01	0.558	-.004	0.002	.074	44.16
affective disorder*	0.256	-.088	0.160	.584	< 0.01	0.286	-.006	0.150	.969	< 0.01
anxiety disorder*	**0.161**	**.400**	**0.147**	**.006**	**25.84**	0.283	.006	0.198	.975	< 0.01
PTSD*	0.240	-.005	0.163	.977	< 0.01	0.238	.273	0.159	.087	16.12
F 54*	0.283	-.181	0.142	.202	0.40	0.346	-.197	0.124	.114	16.34
design: no RCT*	0.249	-1.030	0.642	.108	2.80	0.285	-.128	0.572	.823	< 0.01
length of intervention HPT*^d^	0.291	< .001	0.006	.966	< 0.01	0.261	.002	0.007	.808	< 0.01
number of sessions HPT*^d^	0.197	.003	0.005	.604	< 0.01	0.300	-.001	0.005	.854	< 0.01
Comparator: CBT*	**-0.357**	**.638**	**0.236**	**.007**	**21.03**	-0.029	.321	0.370	.386	< 0.01
number of sessions CG*^d^	0.154	.006	0.006	.348	< 0.01	0.201	.006	0.007	.436	< 0.01

*Note.* Grey shadows for main (i.e. pre-specified) moderators. Significant results of moderator analyses (*p* < .05) are shown in bold. RoB = risk of bias; CG = control group; CBT = cognitive behavioural therapy; NA = moderator analysis could not be conducted due to insufficient variance in moderator. Example for interpretation of a categorical predictor: In studies with no or unclear allegiance against HPT the difference between HPT approaches and other forms of psychotherapy in levels of symptom severity at follow-up is significantly reduced (β = -0.208, *p* = .044). Example for interpretation of a metric predictor: In studies with one percentage more women the difference between HPT approaches and other forms of psychotherapy in levels of symptom severity at end of intervention is not significantly increased (β = < .001, *p* = .867). ^a^*N* varies for individual moderator analysis as studies with missing data in moderators were removed from metaregression analyses. ^b^One study (Dietz et al., 2015) was identified as an outlier and removed from all further meta-analysis. ^c^One study (Herschbach et al., 2010) was identified as an outlier and removed from all further meta-analysis. ^d^metric predictor (all other predictors are dichotomous). *A posteriori analyses.

Exploratory subgroup analyses indicated that the difference between HPT and other psychotherapies regarding symptom severity increased significantly favouring other psychotherapies when CBT was used as comparator and in studies examining anxiety disorders. Sensitivity analysis for this comparison showed that results were not influenced by the study design.

For the outcome *symptom severity at follow-up* subgroup analyses indicated that the difference between HPT in comparison to other psychotherapies was reduced significantly in studies with no or unclear allegiance against HPT (β = -0.208, *p* = .044; for examples for interpretation, please see table note).

Results for secondary outcome domains are shown in Table 1B. For the outcome *interpersonal problems* subgroup analyses indicated that the difference between HPT in comparison to other psychotherapies was reduced significantly in studies with no or unclear allegiance against HPT. Also, the difference between HPT in comparison to other psychotherapies was reduced when bona fide HPT was used, in studies with a low risk of bias due to missing data and in studies not examining anxiety disorders. Yet, somewhat contradictory, the difference increased in favour of other psychotherapies when the control group was not adherent to intervention. No significant moderator effects were detected for the outcomes *general functioning* and *quality of life*.

**Table 1B:** Moderators Analysis for Secondary Outcome Domains

Moderators^a^	Interpersonal problems (*N* = 9^b^)					General functioning (*N* = 12)					Quality of life (*N* = 8)^c^				
	Intercept	β	*SE*	*p*	*R*^2^ in %	Intercept	β	*SE*	*p*	*R*^2^ in %	Intercept	β	*SE*	*p*	*R*^2^ in %
No or unclear allegiance against HPT	**0.502**	**-.519**	**0.169**	**.002**	**> 0.99**	-0.275	.331	0.224	.140	7.22	-0.378	.280	0.171	.102	87.49
Non or unclear bona fide HPT	**0.027**	**.568**	**0.197**	**.004**	**> 0.99**	0.084	-.335	0.235	.154	2.40	-0.098	-.280	0.171	.102	87.49
Non or unclear bona fide CG	NA	NA	NA	NA	NA	NA	NA	NA	NA	NA	NA	NA	NA	NA	NA
Non adherence HPT	0.013	.353	0.231	.127	18.77	-0.165	.069	0.239	.772	< 0.01	-0.099	-.234	0.163	.151	> 0.99
Non adherence CG	**0.071**	**.830**	**0.290**	**.004**	**> 0.99**	-0.100	-.170	0.320	.596	< 0.01	-0.090	-.290	0.170	.079	> 0.99
Low RoB RP	**0.028**	**.362**	**0.169**	**.032**	**> 0.99**	0.036	-.275	0.227	.226	8.16	-0.137	-.104	0.221	.639	< 0.01
Low RoB AI	0.075	.288	0.270	.285	< 0.01	-0.026	-.214	0.231	.353	< 0.01	-0.363	.213	0.204	.296	22.38
Low RoB MI	**0.404**	**-.408**	**0.163**	**.012**	**> 0.99**	-0.037	-.166	0.240	.489	< 0.01	-0.214	-.003	0.211	.987	< 0.01
Low RoB MO	0.254	-.120	0.272	.659	< 0.01	-0.047	-.140	0.240	.559	< 0.01	-0.280	.081	0.256	.752	< 0.01
Low RoB SR	0.157	.063	0.278	.819	< 0.01	-0.105	-.086	0.273	.753	< 0.01	-0.249	.108	0.205	.597	< 0.01
Client centered psychotherapy	-0.102	.391	0.229	.088	41.74	0.016	-.176	0.314	.576	< 0.01	-0.165	-.084	0.211	.691	< 0.01
Population: children	0.143	.196	0.333	.555	< 0.01	-0.120	-.048	0.322	.882	< 0.01	NA	NA	NA	NA	NA
Age*^d^	0.298	-.004	0.012	.748	< 0.01	-0.102	-.001	0.012	.947	< 0.01	-0.384	.004	0.011	.729	< 0.01
% of women*^d^	0.721	-.008	0.007	.238	14.74	0.003	-.002	0.005	.646	< 0.01	-0.558	.005	0.005	.321	< 0.01
Affective disorder*	0.273	-.290	0.263	.270	< 0.01	-0.185	.291	0.293	.322	< 0.01	NA	NA	NA	NA	NA
Anxiety disorder*	**0.071**	**.829**	**0.291**	**.004**	**> 0.99**	-0.081	-.317	0.318	.318	0.05	-0.180	-.290	0.308	.346	8.89
PTSD*	0.229	-.191	0.292	.514	< 0.01	-0.020	-.395	0.233	.091	24.76	-0.215	-.005	0.222	.981	< 0.01
F 54*	NA	NA	NA	NA	NA	-0.133	.063	0.446	.888	< 0.01	-0.264	.093	0.202	.645	< 0.01
Design: no RCT*	NA	NA	NA	NA	NA	NA	NA	NA	NA	NA	NA	NA	NA	NA	NA
Length of intervention HPT*^d^	0.237	.003	0.016	.855	< 0.01	-0.063	-.002	0.010	.801	< 0.01	-0.434	.005	0.008	.552	< 0.01
Number of sessions HPT*^d^	0.341	-.008	0.007	.274	< 0.01	-0.417	.013	0.007	.057	26.08	-0.278	.005	0.011	.665	< 0.01
Comparator: CBT*	NA	NA	NA	NA	NA	NA	NA	NA	NA	NA	NA	NA	NA	NA	NA
Number of sessions CG*^d^	0.243	-.001	0.016	.938	< 0.01	-0.335	.008	0.013	.536	< 0.01	-0.261	.004	0.009	.691	< 0.01

*Note.* Grey shadows for main (i.e. pre-specified) moderators. Significant results of moderator analyses (*p* < .05) are shown in bold. RoB = risk of bias; CG = control group; CBT = cognitive behavioural therapy; NA = moderator analysis could not be conducted due to insufficient variance in moderator. Example for interpretation: In studies with no or unclear allegiance against HPT the difference between HPT approaches and other forms of psychotherapy in levels of symptom severity at follow-up is significantly reduced (β = -0.208, *p* = .044). ^a^N varies for individual moderator analysis as studies with missing data in moderators were removed from metaregression analyses. ^b^One study (Dietz et al., 2015) was identified as an outlier and removed from all further meta-analysis. ^c^Quality of Life: higher score for better QoL. ^d^metric predictor (all other predictors are dichotomous). *A posteriori analyses.

The results for some of the outcome measures should be interpreted with caution because of the small number of studies (*n* = 9 for interpersonal problems, *n* = 12 for general functioning, *n* = 8 for quality of life) in comparison to the large number of moderator analyses.

To test whether primary moderators were sufficiently independent, we examined their associations post-hoc via Fisher’s Exact Test (FET). Results showed a significant association between allegiance and bona fide HPT (*p* = .01, FET). Moreover, bona fide HPT was significantly associated with adherence HPT (*p* = .007, FET) as well as with adherence to the control condition (*p* = .02, FET). There were no other significant associations between primary moderators.

## Discussion

The purpose of this study was to investigate the effects of allegiance and treatment quality (bona fide psychotherapy, adherence) for assessing the comparative effectiveness of psychotherapy. We used data comparing the effects of HPT subapproaches (from a study pool used in a recent evaluation of the GSBP) to other evidence-based treatments.

Moderator analyses did not indicate consistent effects of allegiance, treatment quality or study quality on the comparison between HPT and other forms of psychotherapy across all outcomes and assessment points. At the end of intervention, we found a moderating effect in one *(interpersonal problems*) out of four outcomes (*symptom severity, general level of functioning, quality of life*) for allegiance (no or unclear against HPT subapproach) and treatment quality (non bona fide HPT subapproach, non-adherence to control condition) and – to a somewhat lesser extent – also for study quality. At follow-up (with only one outcome domain still available: symptom severity), allegiance significantly moderated comparative treatment effects: in studies with no allegiance against HPT the difference between HPT and other forms of psychotherapy was significantly reduced for *symptom severity at follow-up*. The beta coefficient for symptom severity at end of intervention is pointing in the same direction (ß = -0.183) as at follow-up (ß = -0.208) but is not as pronounced in size.

Exploratory subgroup analyses showed that the difference between subapproaches of HPT in comparison to other psychotherapies was reduced in studies *not* examining anxiety disorders and in studies *not* using CBT as a comparator.

### Allegiance

The high proportion of *allegiance* against HPT as well as the high number of non-bona fide treatments in the HPT-treatment arm in the present meta-analysis has also been reported in other studies (Cuijpers et al., 2012; Elliott et al., 2013; Elliott et al., 2021). Cuijpers et al. (2012) concluded that non-directive supportive therapy (as a subapproach of HPT) for depression is equally effective as other psychological treatments after researcher allegiance was controlled. Similarly, Elliott et al. (2013) report a drop in (weighted) effects when comparing supportive therapies to other psychotherapy approaches. In addition, researcher allegiance has been demonstrated to have an impact on outcomes in psychotherapy studies not focusing on HPT alone (Munder et al., 2013).

When focusing on the impact of allegiance, it is important to keep in mind that it is not useful to interpret it as a purposeful attempt to skew results but rather take it into account as a human tendency to believe in one’s own ideas and practices in a way that objectivity is compromised (Lomangino, 2016; Yoder et al., 2019). This perspective is supported by research that shows that awareness and acceptance of its potential impact may reduce its effects (Munder et al., 2013). Another option to avoid a bias through allegiance are allegiance-controlled trials where the interventions in the different conditions are planned and supervised by proponents of the respective approaches (Barkham et al., 2021; Leichsenring et al., 2009). Similarly, more recently, bona fide is also explicitly considered in trial designs: In their recent RCT comparing person-centered experiential therapy (PCET as a HPT subapproach) and CBT, Barkham et al. (2021) carefully implemented bona fide treatment arms with a similar level of professional training with the help of trainers qualified in the respective interventions.

### Adherence

We did not find moderation effects of adherence on comparative effectiveness. A recent systematic review and meta-analysis of the role of adherence for outcome in child and adolescent psychotherapy found a small (statistically significant) (interventional, non-comparative) effect size for adherence of *d* = 0.096 (95% CI [.058, .124]. Considering the very small effect size, the authors conclude - despite the statistical significance - that other factors than adherence are much more relevant to consider for outcome. They included primary studies if “external or independent observer rated measures of adherence or competence, measures across multiple time points (…)., and interrater agreement established in the study or use of coders trained to this level (…) (ICC) > -0,60 or Kappa > -0,61 or percent agreement > - 90% or the score is based on agreement by multiple rates” (Collyer et al., 2020, p. 419). Our criteria to evaluate adherence were less demanding than in Collyer et al. (2020). When adherence as a construct is significantly linked to outcome, quite likely its actual impact upon explaining differences in outcomes remains very small with the presented mean effect size estimation of *r* < 0.10 (i.e. accounting for less than 1% of the variance in therapy outcome).

### Study Quality

With respect to *study quality* (risk of bias, assessed via RoB), we found no clear evidence for moderation effects. These results are in line with the recent studies by Cuijpers, Quero, et al. (2021) as well as Hoppen and Morina (2020). In this context it has to be kept in mind that study quality is quite likely not fully independent from allegiance: Munder et al. (2011) found that allegiance is more strongly related to outcome in studies with lower methodological quality. The inclusion criteria of the GSBP define relatively high methodological standards. These standards may have prevented that allegiance effects unfold as strongly in our study as maybe in other meta-analyses where inclusion criteria are less methodologically strict.

### Strengths and Limitations

The present study compasses a considerable number of trials, examined important predefined moderators and demonstrates the challenges in evaluating the significance of allegiance and treatment quality (bona fide and adherence). However, in conducting this meta-analysis, we also were faced with a number of problems:

#### Limitations: Allegiance

Allegiance was defined via an aggregate score taking into account researchers’ allegiance, therapists’ allegiance, trainers’ allegiance and supervisors’ allegiance was used (Steinert et al., 2017). This aggregation forecloses to identify which type of allegiance may be more (or less) relevant for treatment outcome.

#### Limitations: Treatment Quality

Following Benish et al. (2008) and Wampold et al. (1997) we used a sophisticated bona fide rating scheme with five criteria: use of a manual; application of an intervention based on psychological principles; author(s) indicate(s) affiliation with the examined intervention (sub)approach on own website; author has other publications concerning the examined intervention (sub)approach; therapists received a training (Benish et al., 2008; Wampold et al., 1997). We decided to use a liberal categorization with only two of the above aspects necessary in order to categorize the intervention as an overall bona fide treatment. Despite these seemingly clear criteria, the assessment of bona fide turned out to be challenging because of often insufficient information.

Further difficulties arose concerning the manual criterion. Many studies reported the use of a manual, also in the HPT condition. However, referenced manuals differed considerably in terms of accessibility, comprehensiveness as well as in terms of content (*common* manuals vs. review or overview articles). This constitutes a general problem in psychotherapy research and practice, affecting not only HPT (Willutzki & Andermann, 2019). Therefore, extensive discussions about the manual criterion were necessary, and decisions on the categorization are sometimes not clear cut. For rating adherence, all following criteria had to be fulfilled: treatment conditions needed to be described precisely; the exact (sub)approach must be presented in detail; adherence had to be rated by external observers. Related to the latter, studies sometimes indicated that adherence was rated by external observers but the actual result was missing. In these cases, we decided to rate the criterion of adherence liberally, even if authors did not report their adherence results.

#### Further Limitations

The most common diagnostic groups of the systematic review were psychological and behavioural factors associated with disorders or diseases classified elsewhere (F54). The GSBP used this category for a wide scope of applications. Thus, there were also studies included examining subjects with (*only*) a diagnosis of a somatic illness (e.g., patients with age-related macular degeneration, Rovner et al., 2014).

While we based our analysis on the 114 studies taken into account by the GSBP it is important to note that our study pool for the moderator analysis is not identical to the study pool underlying the differentiated GSBP-evaluation of HPT (GSBP, 2018). Discrepancies are due to differences between the method paper (GSBP, 2010) and our study protocol. As an example, the GSBP excluded some of the 114 studies from further analysis due to insufficient methodological quality. However, studies with low methodological quality were included in the present meta-analysis as study quality was one of the moderators by using Cochrane’s risk of bias or ROBIN-I tool. On the other hand, we had to exclude 19 studies for which either the study design was not appropriate for the present research question (e.g., HPT subapproach vs. other HPT subapproach or HPT subapproach mixed with other psychotherapy approach), or no mean values were given needed to conduct statistical analyses going beyond the evaluation of the GSBP.

As in many meta-analyses trying to explore heterogeneity among studies via subgroup analyses we face power problems, particularly because studies are not evenly distributed between subgroups. It is still useful and necessary to run the respective subgroup analyses. On the other hand, one could also argue that experiment wise error rates may assist in arguing that the significant results may have occurred by chance alone in the absence of any true effects. Further, it has to be kept in mind that no evidence of the moderators’ impact does not mean that this is evidence of no difference (Cuijpers, Griffin, & Furukawa, 2021). Finally, most of the Humanistic studies were studies based on Rogerian psychotherapy and thus results are particularly relevant for this kind of interventions.

### Conclusion

The present results could not demonstrate effects of the examined moderators on treatment outcome. However, we mainly examined studies with high study quality. This is crucial to consider for generalizing our results and it is necessary to prove whether results can be replicated for studies with low study quality.

## Supplementary Materials

The Supplementary Materials contain the following items:

- **Study protocol:** Schünemann et al. (2019)
- **Online appendices:** Schünemann et al. (2025S):
  - ***Appendix A – Supplementary Tables***
    - *Supplementary Table 1.* Study characteristics (*N* = 50)
    - *Supplementary Table 2.* Study quality (Cochrane’s risk of bias tool for RCT or ROBIN-I for non-RCT); Allegiance, Bona fide and Adherence rating (*N* = 50)
    - *Supplementary Table 3.* Different outcome domains for the comparison humanistic psychotherapy approaches vs. other psychotherapies
  - **Appendix B – Supplementary Figure**
    - *Supplementary Figure 1.* Exemplary funnel plot of the comparison HPT approaches vs. other psychotherapy only for the primary outcome symptom severity at end of intervention
  - **Appendix C – Supplementary Information**
    - *The evaluation procedure of the GSBP*
    - *Additional inclusion and exclusion criteria*
    - *Preliminary Results: Overall main effect sizes of HPT*

SchünemannO.
JansenA.
WillutzkiU.
HeinrichsN.
 (2025S). Supplementary materials to "Allegiance and treatment quality as moderators of the comparative effectiveness of psychotherapy? A systematic review and meta-analysis of studies comparing humanistic psychotherapy to other psychotherapy approaches"
[Online appendices]. PsychOpen. 10.23668/psycharchives.15954
PMC690212831763989

## Data Availability

The set of extracted data is available upon request.

## Reporting Guidelines

The study followed the Preferred Reporting Items for Systematic reviews and Meta-Analyses (PRISMA; Page et al., 2021).
